# Educational inclusion and satisfaction of families of students with intellectual disabilities: a bibliometric study

**DOI:** 10.3389/fpsyg.2024.1335168

**Published:** 2024-03-22

**Authors:** Susana Tebar-Yébana, Diego Navarro-Mateu, María Teresa Gómez-Domínguez, Valentina Gómez-Dominguez

**Affiliations:** ^1^Doctoral School, Catholic University of Valencia San Vicente Mártir, Valencia, Spain; ^2^Department of Specific Educational Needs and Attention to Diversity, Faculty of Education Sciences, Catholic University of Valencia San Vicente Mártir, Valencia, Spain; ^3^Faculty of Education Sciences, International University of Valencia, Valencia, Spain

**Keywords:** satisfaction, families, disability, inclusive education, bibliometrics

## Abstract

This bibliometric study scrutinizes the corpus of scientific output within the Web of Science pertaining to familial satisfaction among parents raising children with intellectual disabilities, focusing specifically on the milieu of educational inclusion. The analysis discerns a discernible ascension in scholarly interest in this domain, encapsulating 77 papers emanating from 75 journals, incorporating an aggregate of 3,497 cited references. Our investigation delineated 354 researchers across 39 nations, underscoring the transnational purview of this scholarly endeavor. The United States emerged as the pre-eminent contributor, with Canada and the United Kingdom following suit. Collaboration on an international scale was notably led by the US, with the UK and Australia trailing in tandem. Prominent institutions were identified for their scholarly output; the University of Kansas led with four papers, followed closely by Monash University, University of California Los Angeles, and University of California Riverside, each contributing three papers. Of particular note, the University of Kansas accrued 250 global citations (TGCS). A total of 75 journals were encompassed in this study. The Journal of Intellectual Disability Research emerged as the vanguard with four published papers, closely trailed by Child Care Health and Development and Exceptional Children. Notably, the latter boasted the highest impact factor (JCR = 4.09; Q1). In summation, this review proffers a meticulous and expansive overview of extant scholarship concerning the experiences of families rearing children with disabilities within the inclusive education framework.

## 1 Introduction

The United Nations General Assembly (2006) approved the Convention on the Rights of Persons with Disabilities (ONU, 2006). In article 24, the Convention establishes the right of persons with disabilities to receive education on the basis of equal opportunity. Pursuant to the Convention, the inclusive education approach has gradually increased in importance, and the significance of guaranteeing equitable opportunities for all students has been acknowledged (Moreno and Molins, 2020).

In terms of both national and international legislation, progress has been made in the formulation of regulations which guarantee inclusive and high quality education that ends any form of exclusion. This progress toward educational inclusion also entails greater recognition of the importance of the effective participation in educational processes by the educational community as a whole, and the role played by families in ensuring equitable and quality education becomes fundamental (Moreno and Molins, 2020).

As a result, this movement for inclusion has become a project for social and public participation which calls for significant changes in schools to ensure that no student is excluded from the system, which means that schools must take steps to enable the entire educational community – adults and schoolchildren – to participate in the life of the school (Booth and Ainscow, 2015; Silveira, 2016; Arnaiz, 2019). Education which is based on the principles of equality and equity must therefore take families into account, and families with children with disabilities must be involved in the processes of education, and their perception of their participation in school life and their satisfaction with their children’s educational inclusion must be ascertained (Knoche et al., 2006; Meleady et al., 2018). Consideration should also be given to the fact that the family system is a factor for protection for students with special educational needs, which must not be overlooked by schools (Bellin et al., 2010; Sánchez-Pujalte et al., 2020).

However, recognition of the family’s right to participate in school life provides no guarantee that it will be exercised and implemented under optimal and satisfactory conditions. It is still considered a right that is exercised to a limited extent in practice, with a significant divide between teachers and families who have children with disabilities (Silveira, 2016). Echeita (2017) highlights a clear contradiction between the provisions of the legislation and the real situation in many schools, which leads to tension and emotional difficulties that have a negative effect on vulnerable students and their families.

Accordingly, it is also necessary to take into account the evidence showing how families with children with disabilities report a significantly lower quality of life and lower levels of family satisfaction than children without disabilities, and how this has a negative on their children’s cognitive development and functioning in their educational environment (Ridosh et al., 2016). Families explicitly state that they feel they do not feel that they belong to the school where their children are enrolled, and identify discriminatory attitudes (Wilkins et al., 2010; Hsieh et al., 2013; Padilla et al., 2023). They also say that having a child with an intellectual disability has a strong impact, which leads to poorer family functioning and a perceived lower level of satisfaction with the educational support services received, which has been further aggravated by the recent health situation arising from the COVID-19 pandemic (Neely-Barnes et al., 2008; Davies and Beamish, 2009; Al-Krenawi et al., 2011; Murphy et al., 2021).

At this point, and within the regulatory framework governing educational inclusion, schools must avail themselves of participation by the families when planning educational pathways for students with disabilities, and establish clear objectives that foster inclusive and high quality education. Accordingly, in order to contribute to improving families’ satisfaction in the educational context that facilitates this cooperation, DeChillo et al. (1994), propose four significant dimensions that should be taken into account in educational planning to foster the participation process. Those dimensions are the supportive relationships that are established, the agreements reached, a forthright exchange of information, and a flexible and shared approach in the cooperation between professionals and families.

So, in contributing to the improvement of inclusive education (Moore et al., 2018), the need to understand the level of satisfaction of families regarding the educational inclusion of their children becomes evident. Hence arises the research question of the present study: What is the current state of academic production and, therefore, the existing interest in the global scientific community regarding the satisfaction of families with children with disabilities toward the response offered by the school system from an inclusive perspective? All of this considering empirical studies indexed in the Web of Science Core Collection and using a bibliometric approach for it. According to Woods et al. (2018), investigating family satisfaction contributes to promoting greater communication and participation in the educational environment, which is essential for improving educational quality and inclusion. Similarly, Schenker et al. (2017) state that collaboration between families and educational institutions is a key factor positively influencing family satisfaction.

Taking all of the above into account, this study aims to contribute to the enhancement of inclusive education, and offer an exhaustive bibliometric study as a valuable tool for examining and understanding the scientific literature related to the satisfaction with educational inclusion among families with children with intellectual disabilities. As previously described, it is important to take the close relationship between family satisfaction and inclusive practices into account, and as such this research will consider the area of interest described in depth, identifying the main areas of research, emerging trends and gaps in scientific knowledge (Zanobini et al., 2017). Likewise, the findings supported by research conducted by the most important authors in the field can contribute to improving our understanding of these families’ needs and challenges, and provide a solid foundation for future research and work in the area of study defined (Ardanuy, 2012; Davey et al., 2015). The objective is to promote educational inclusion, enhance the quality of family life and facilitate the development of students with disabilities to the greatest extent possible (Brown et al., 2010).

## 2 Materials and methods

### 2.1 Data collection

This bibliometric study examines scientific production concerning the satisfaction of families with children with disabilities with educational inclusion by searching the Web of Science (WoS) Main Collection. The database selected is a reliable source containing the major bibliometric indicators, a wide range of specialized indexes which are grouped by subject or content indexing (Pranckutė, 2021). An analysis based on a bibliometric approach helps to organize the information, perform the most relevant selection and establish categories in order to consider the information in quantitative and qualitative terms (Gallegos et al., 2020).

The data collection began in March 2023, and in order to meet the objective, the quality indicators established by the PRISMA 2020 approach were followed in order to obtain a valuable review of the field of study and to ensure that relevant information was captured (Page et al., 2021).

An advanced search by subject was carried out by referring to the title, abstract or keywords of the articles. The search string used in the subject field was as follows:

((“Famil* satisfaction*”) or (Famil* near/5 satisfaction*) or (“pleasure* famil*”) or (“famil* contentment”) (Subject) and (“Intellectual* Disabilit*”) or (disabilit*) or (special* need*) or (“Intellectual* Disabilit*” near/5 children*) (Subject) and *School* or (“*School inclusion*”) or (“inclusive education*”) or (“*education inclusion*”))

The total number of articles obtained was 172. Subsequently, some were excluded due to duplication (*n* = 1), as well as based on criteria set by automation tools, such as open access availability and/or originating from different databases (*n* = 10), leaving a total of 161 articles to which the different inclusion and exclusion criteria were applied. The inclusion criteria were as follows: (1) literature reviews and empirical studies (qualitative and quantitative); (2) scientific journal articles; (3) published in any language in the last 5 years; (4) in the main collection of Web of Science; and (5) families with children with special educational needs, assessing their satisfaction with the educational system’s response. This led to the selection of 112 articles.

**Figure 1:**
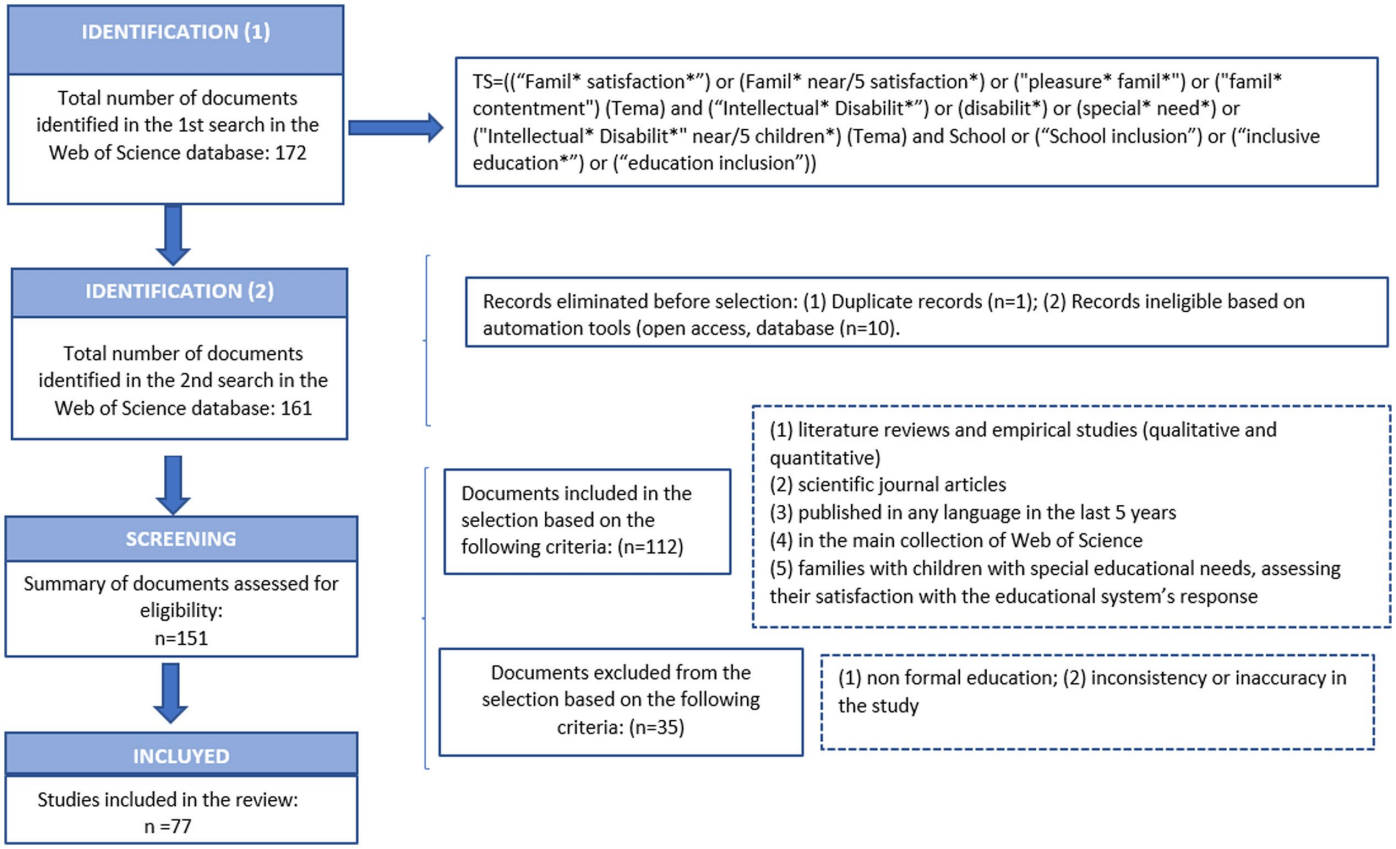
PRISMA flowchart detailing the steps in source identification and selection. Adapted from Thananusak (2019) and Page et al. (2021).

Subsequently, after reviewing the content of these articles, the following exclusion criteria were applied: (1) non formal education; (2) inconsistency or inaccuracy in the study. This led to the exclusion of 35 articles and thus to the selection of a total of 77 articles (Figure 1).

Finally, unknown or incomplete data, author names, misspellings, initials, synonyms and homonyms (Jensen et al., 2009) were reviewed using different resources such as the author’s address or even Google to identify data that did not appear in the WoS database. In this sense, the review of institutions was also carried out, as changes were observed in some of them, and the most current one was chosen (Giménez-Espert et al., 2019).

### 2.2 Bibliometric analysis

The statistical software used and the analyses performed with each one are presented below: HistCite software (version 2010.12.6; HistCite Software LLC, New York, NY, USA) (Padrón and Pirela, 2017): Several basic bibliometric indices were calculated using this program, covering the number of articles per year, author, country, institution and journal. HistCite provides a clear presentation of information and lists quality indicators, such as the total global citation score (TGCS) and the total local citation score (TLCS). The TGCS shows the total number of citations received by the articles analyzed, while the TLCS shows the number of citations received in the Web of Science (WoS) database, for only the articles selected in the specific analysis performed. This software not only provides bibliometric indices and quality scores, but also provides a historical analysis of citations, displays citation networks, identifies collaboration patterns and can be customized. These additional features make HistCite a comprehensive and versatile tool for bibliometric analysis (Wulff-Barreiro, 2007).

VOSviewer software (Van Eck and Waltman, 2017): VOSviewer is a program used for bibliographic and thematic linkage analysis. It enables users to examine the connections between articles, and to see bibliometric networks. One of the major advantages of VOSviewer is its ability to create clusters, showing the similarity between two or more items based on the number of references they have in common. This software is particularly useful in systematic literature reviews, as it is not influenced by when the analysis is performed. It also facilitates identification of significant articles in a given field of study. For all these reasons, this program is particularly useful due to its ease of use and interactive display, and allows data to be explored and examined dynamically (Viner et al., 2020).

R bibliometric software package (Derviş, 2019; Aria et al., 2020): Several analyses were performed using the bibliometric package in the R programming environment. This program enabled examination of co-authorships, collaborations between countries, and the most common keywords in the articles analyzed. It also enabled thematic analysis in order to discover emerging, current and neglected topics in the field of study. One of the advantages of the R software and its bibliometric package is its flexibility when generating various types of graphs, such as networks, three-field graphs, word clouds, thematic maps, histograms, strategic diagrams, evolution maps and world maps. These graphics display the results of the bibliometric analysis clearly and effectively (Moral-Muñoz et al., 2020).

In short, HistCite provides basic bibliometric indexes and a detailed assessment of the quality of articles, while VOSviewer focuses on bibliographic and thematic linkage analysis, and the R bibliometric software provides comprehensive analyses of co-authorships, collaborations between countries, keywords and emerging themes. Each program has its own specific advantages and characteristics, and together they provide a comprehensive view of scientific production and its impact on the field of study.

## 3 Results

As described above, a total of 77 articles published in 75 journals and written by 354 authors were retrieved after searching the WoS database and performing an exhaustive review. The mean number of citations per document was 16.82. In addition, 292 keywords and 272 author’s keywords were identified. The mean number of authors per document is 4.4, which when rounded up is 5. The international collaboration rate is 23.81%. These data are summarized in Table 1.

**Table 1:** Main information.

Main information about the data	
Timespan	1993:2022
Sources (Journals, Books, etc.)	75
Documents	84
Annual growth rate %	7.87
Document average age	8.32
Average citations per doc	16.82
References	3,497
Document contents	
Keywords plus (ID)	292
Author’s keywords (DE)	272
Authors	
Authors	354
Authors of single-authored docs	2
Authors collaboration	
Single-authored docs	2
Co-authors per doc	4.43
International co-authorships %	23.81
Document types	
Article	77
Article; book chapter	1
Article; early access	2
Article; proceedings paper	1
Review	3

### 3.1 Basic indicators

This first section of results provides some basic indicators. A detailed description of the number of papers and citations per year, the number of papers and citations per author, per institution and per country is presented. It also includes information about journals that published at least one article, indicating the number of publications, citations and their impact factor. Finally, the keywords used by the authors are shown as they relate to the years of publication.

#### 3.1.1 Years

**Figure 2:**
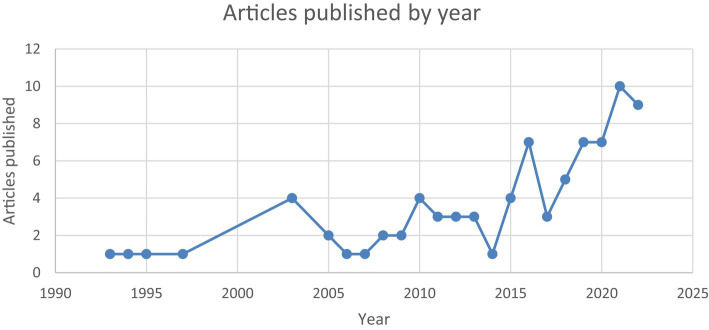
Articles published by year.

The total number of published articles is 77, which were published in the period between 1993 and 2022, as shown in Figure 2. The first article was published in 1993, with a single publication in that year. The number of publications increased over time, and the initial number was surpassed in 2003, when four articles were published. Interestingly, despite fluctuations in the number of articles per year, the overall trend is upward, indicating an increase in the popularity of the research topic.

#### 3.1.2 Authors

A total of 349 researchers were identified, who published at least one article on the topic of satisfaction with educational inclusion among families of students with disabilities.

**Figure 3:**
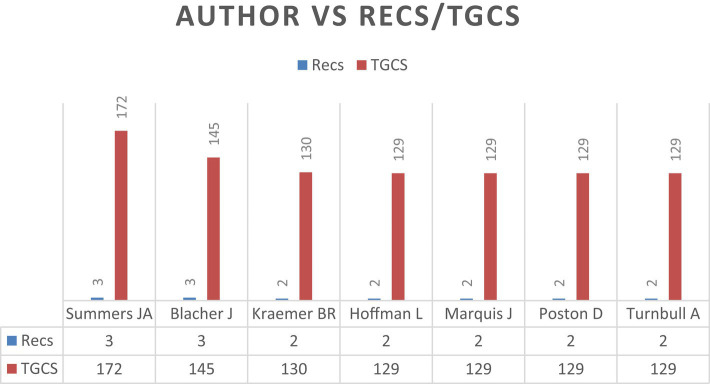
Authors with the most publications and highest TGCS (≥129 TGCS).

The leading researchers in this field include Blacher J and Summers JA, who published 3 papers each. Summers JA, Blacher J and Kraemer BR were among the authors with the most global citations, with 172, 145 and 130 citations, respectively. These results are presented in Figure 3, which shows a comparison of the authors with the most publications and global citations, and establishes a cut-off point of 129 global citations. Information on the Recs (the number of times the author’s papers were cited) and the Total Global Citation Score (the score received by the author’s papers) is also included.

#### 3.1.3 Institutions

**Figure 4:**
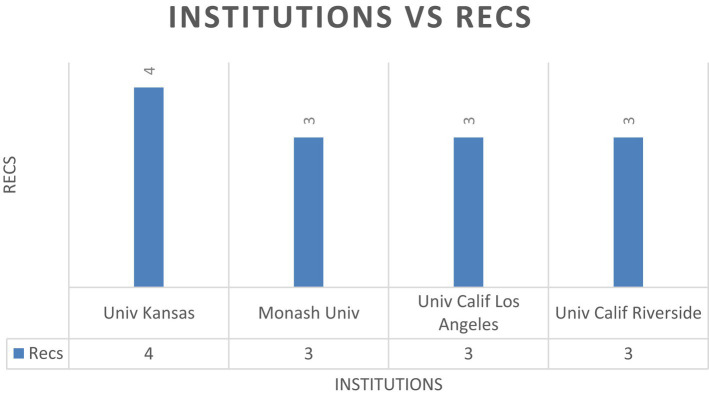
Number of publications by institutions ≥3 Recs. N.B., Recs-number of articles.

As shown in Figure 4, when a cut-off point of 3 publications is established, the following universities have the most documents published: *University of Kansas, Monash University, University of California Los Angeles and University of California Riverside*, with four papers for the first university, and three papers for the others.

**Figure 5:**
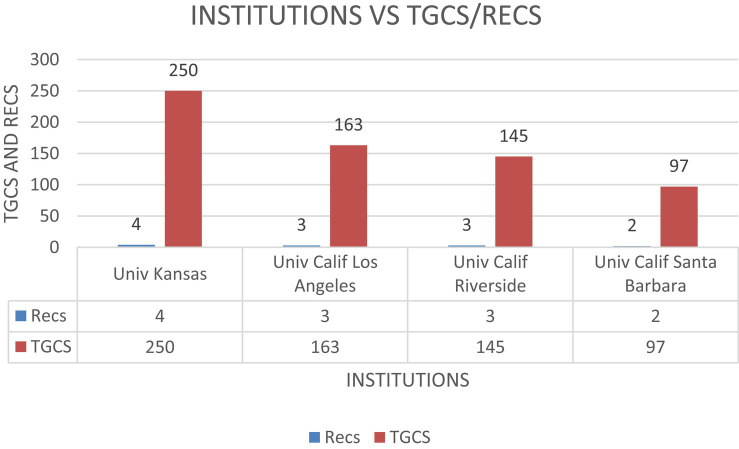
Number of TGCS by institution (≥97 TGCS). N.B., Recs-number of articles; TGCS, Global Citation Score.

However, when global citations are examined and a cut-off point of 97 citations is established, as shown in Figure 5, *University of Kansas* leads the list, with a total of 250 global citations. It is followed by *University of California Los Angeles* with 163 citations, *University of California Riverside* with 145 citations, and finally *University of California Santa Barbara* with 97 citations.

#### 3.1.4 Countries

**Figure 6:**
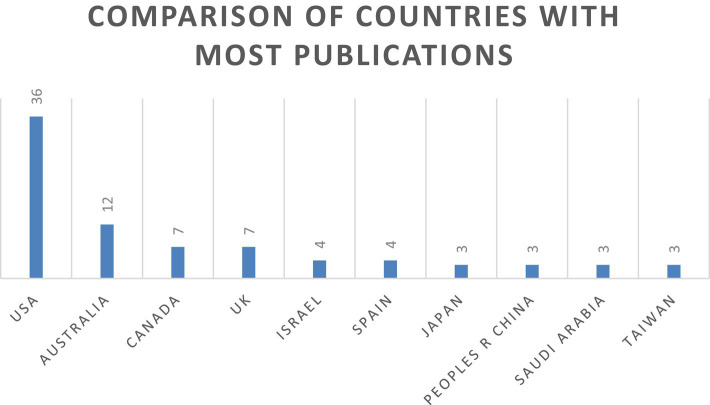
Comparison of countries with the most publications (≥3 Recs).

Researchers from a total of 39 countries published at least one article on the research topic. When a cut-off point of 3 articles is established, the United States is the country with the most publications, with a total of 36. Australia is next with 12 publications, followed by Canada and the United Kingdom with 7 publications each. Israel and Spain have 4 publications each. Finally, Japan, China, Saudi Arabia and Taiwan have published 3 articles each. These data are shown in Figure 6.

After the countries with the most publications have been examined, the order changes when the number of global citations received is taken into account. The United States and Australia continue to head the list, with 964 and 184 global citations, respectively. However, the order of the countries following them changes, and new countries join the list.

**Figure 7:**
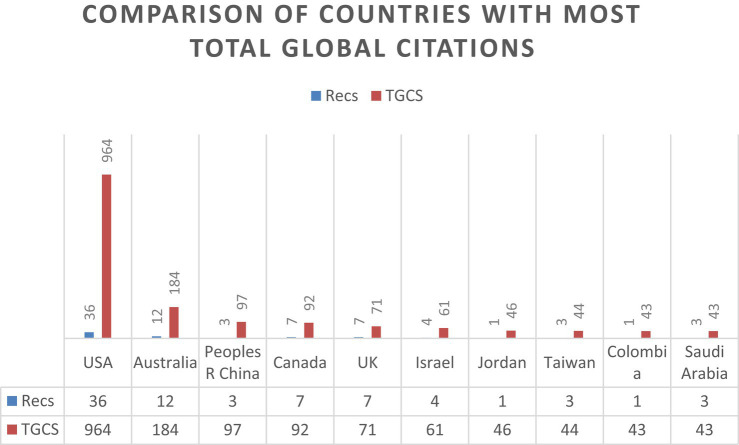
Comparison of countries with the most publications (≥43 TGCS).

The following countries are ranked in order of publications, from the most to the least: China (97 global citations), Canada (92 global citations), United Kingdom (71 global citations), Israel (61 global citations), Jordan (46 global citations), Taiwan (44 global citations), and finally Colombia and Saudi Arabia with 43 global citations each. These data are presented in Figure 7.

#### 3.1.5 Journals

A total of 75 journals published at least one article on the topic studied. Only one of all these journals published four articles, while six journals published two articles each, and this indicator was established as the cut-off point (see Table 2).

**Table 2:** Journals by the number of publications and citations received (TGCS) and impact factor (JCR) (≥2 Recs).

Journal	Recs	TGCS	TGCS/t	Impact factor
Journal of Intellectual Disability Research	4	108	12.38	3.6
Child Care Health and Development	2	41	3.64	2.9
Exceptional Children	2	101	7.78	4,09
Frontiers in Education	2	0	0.00	2.3
Intellectual and Developmental Disabilities	2	83	5.83	2.35
Journal of Applied Research in Intellectual Disabilities	2	51	3.84	2.16
Journal of Pediatric Rehabilitation Medicine	2	12	1.71	1.9

N.B., Recs-number of articles; TGCS, Global Citation Score; TGCS/t, Global Citation Score per year; − means that these journals are in ESCI and therefore do not yet have an impact factor.

Some journals are of interest due to the number of articles published. The *Journal of Intellectual Disability Research* heads the list, with four articles published. It is followed by *Child Care Health and Development*, *Exceptional Children*, *Frontiers in Education*, *Intellectual and Developmental Disabilities*, *Journal of Applied Research in Intellectual Disabilities* and *Journal of Pediatric Rehabilitation Medicine*, which all published two articles.

These figures show the most prominent journals in terms of number of articles published on the research topic.

Meanwhile, as regards the impact factor of these 7 journals that published the most articles, the journal *Exceptional Children* has the highest impact factor (JCR = 4.09; Q1), followed by *Journal of Intellectual Disability Research* (JCR = 3.6; Q1) and in third place by *Child Care Health and Development* (JCR = 2.9; Q2). The results are shown in Table 2.

The journal that published the most articles is also the one that received the most global citations (108) and the most citations per year (12.38). Interestingly, despite having only 2 publications, *Exceptional children* closely follows the *Journal of Intellectual Disability Research* with 101 global citations, although its number of citations per year is much lower in comparison, at only 7.78.

#### 3.1.6 Most common keywords

**Figure 8:**
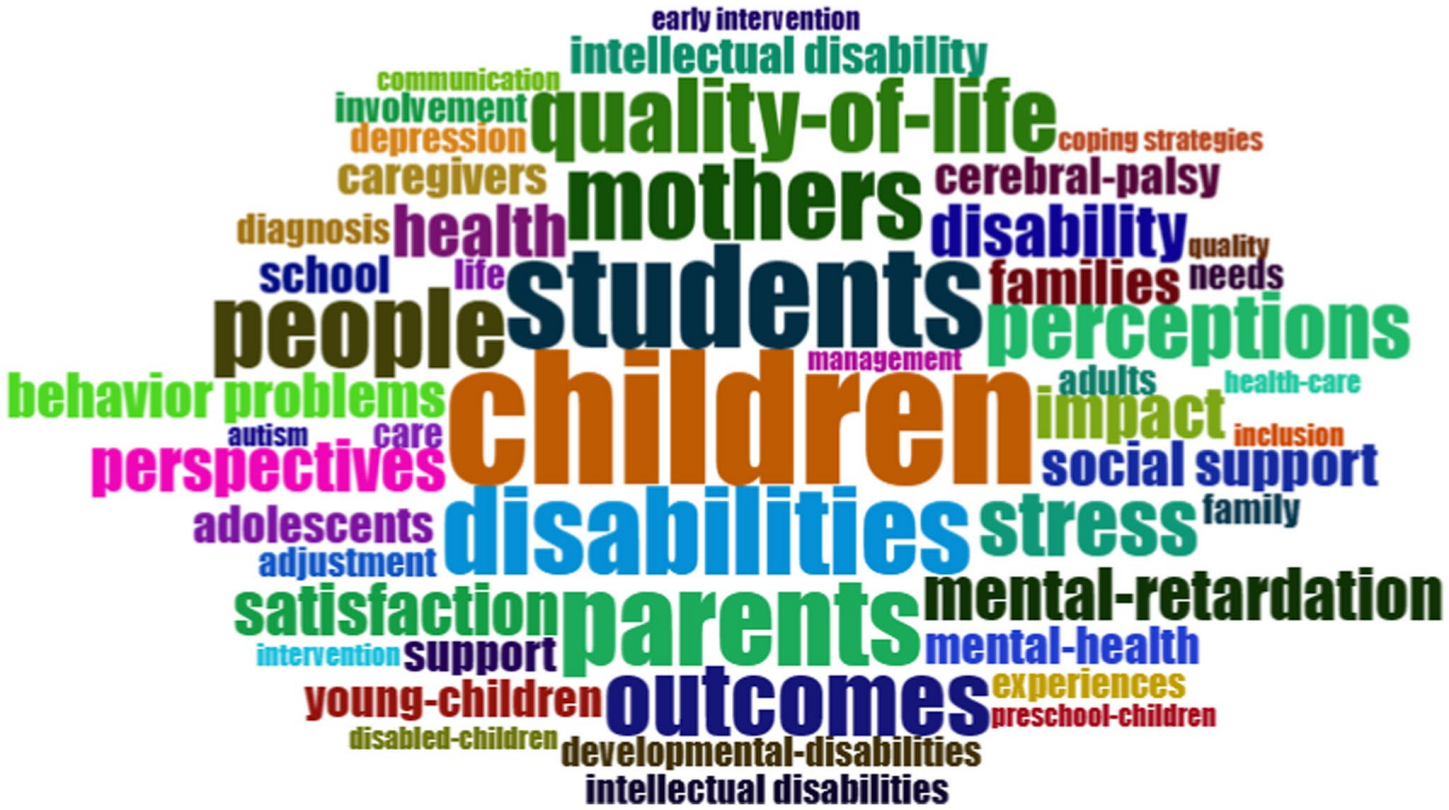
Most common words (≥3 common words).

There are 50 most common keywords used in the publications studied, after establishing a cut-off point of a frequency of 3 or more, and these are presented in Figure 8. The most common terms include *children*, with a frequency of 18 and *students*, with a frequency of 14. These are followed by *disabilities* and *parents*, with a frequency of 12 each. They are followed by *mothers* and *people* with a frequency of 11, and lastly, by outcomes and *quality-of-life* with a frequency of 10 each.

These terms reflect the most relevant and frequently addressed concepts in publications related to the research topic.

### 3.2 Co-citation analysis

A co-citation analysis will be carried out in this section. Co-authorship network will be presented, followed by cross-country collaboration networks and finally, keyword networks will be shown. These results have been displayed and represented on the maps presented below.

Relationships and collaborations between authors are analyzed in the co-authorship map, and the links and patterns of co-authorship identified. This shows the research communities and the links between authors in the field of study.

The collaboration networks between countries are subsequently presented, and international cooperation and the links established between different nations are investigated. This provides an understanding of the global dynamics of research and transnational collaborations on the topic studied.

Finally, the keyword networks are shown, in which the relationships and connections between the terms used in the publications are analyzed. This helps to identify the main topics and areas of focus in the field of study.

#### 3.2.1 Co-authorship

Of the total number of 354 authors, only collaborations between authors who wrote one or more articles together have been presented. This identified a total of 12 co-author networks involving 32 researchers who have published together on this research topic. These networks reflect collaborations between authors and reveal patterns of teamwork in the field.

**Figure 9:**
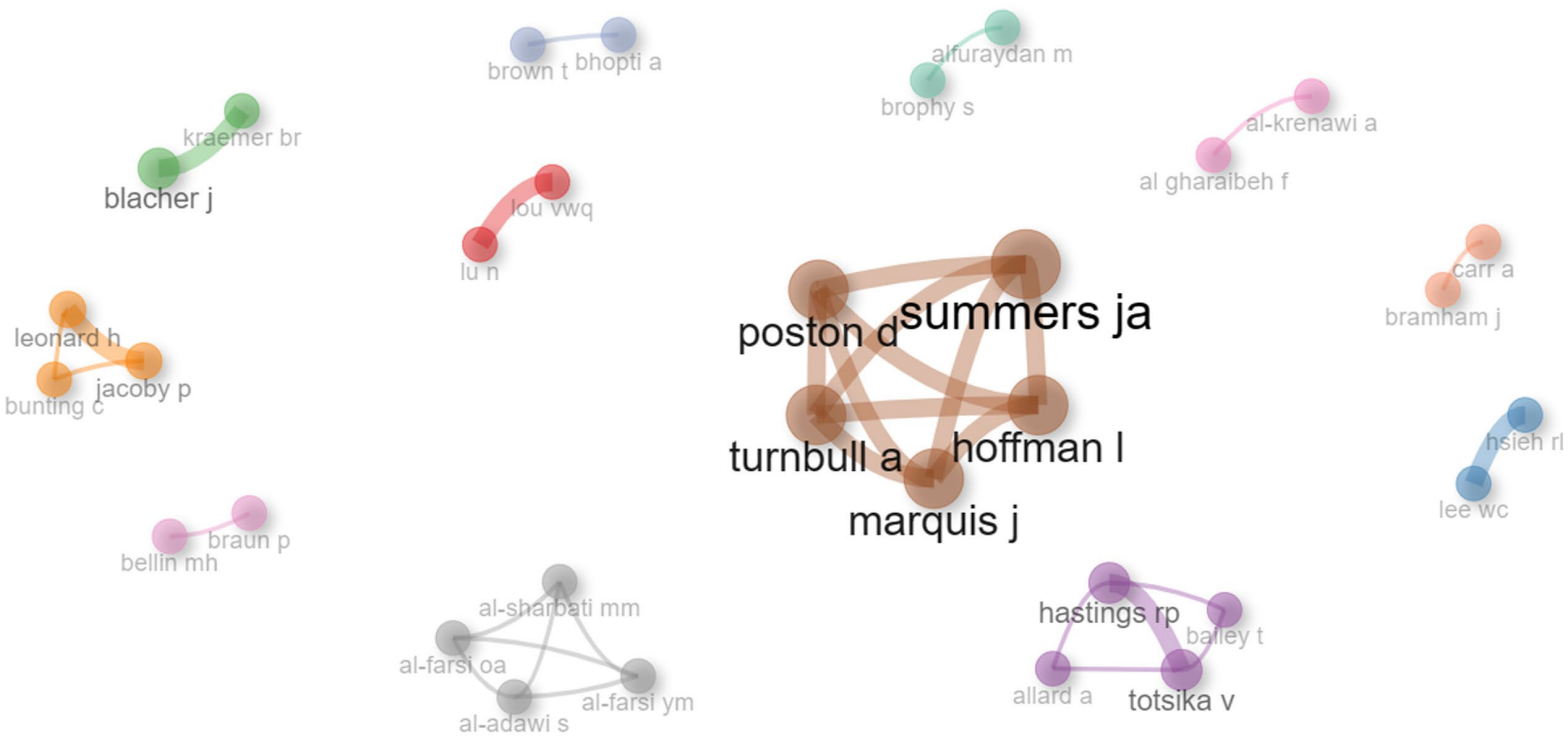
Co-authorship networks (≥1 collaboration).

Figure 9 shows the different collaboration networks, in which the connections between authors can be clearly observed. There is a main network which includes 5 researchers, indicating that they collaborate more closely and more frequently. Two networks of 4 investigators, one network of 3 investigators and eight networks of 2 investigators were also identified.

This analysis provides valuable information on collaboration and interaction between researchers in the field of study. These findings suggest the existence of consolidated research groups, and closer collaborations between some authors in particular.

#### 3.2.2 Collaborations between countries

**Figure 10:**
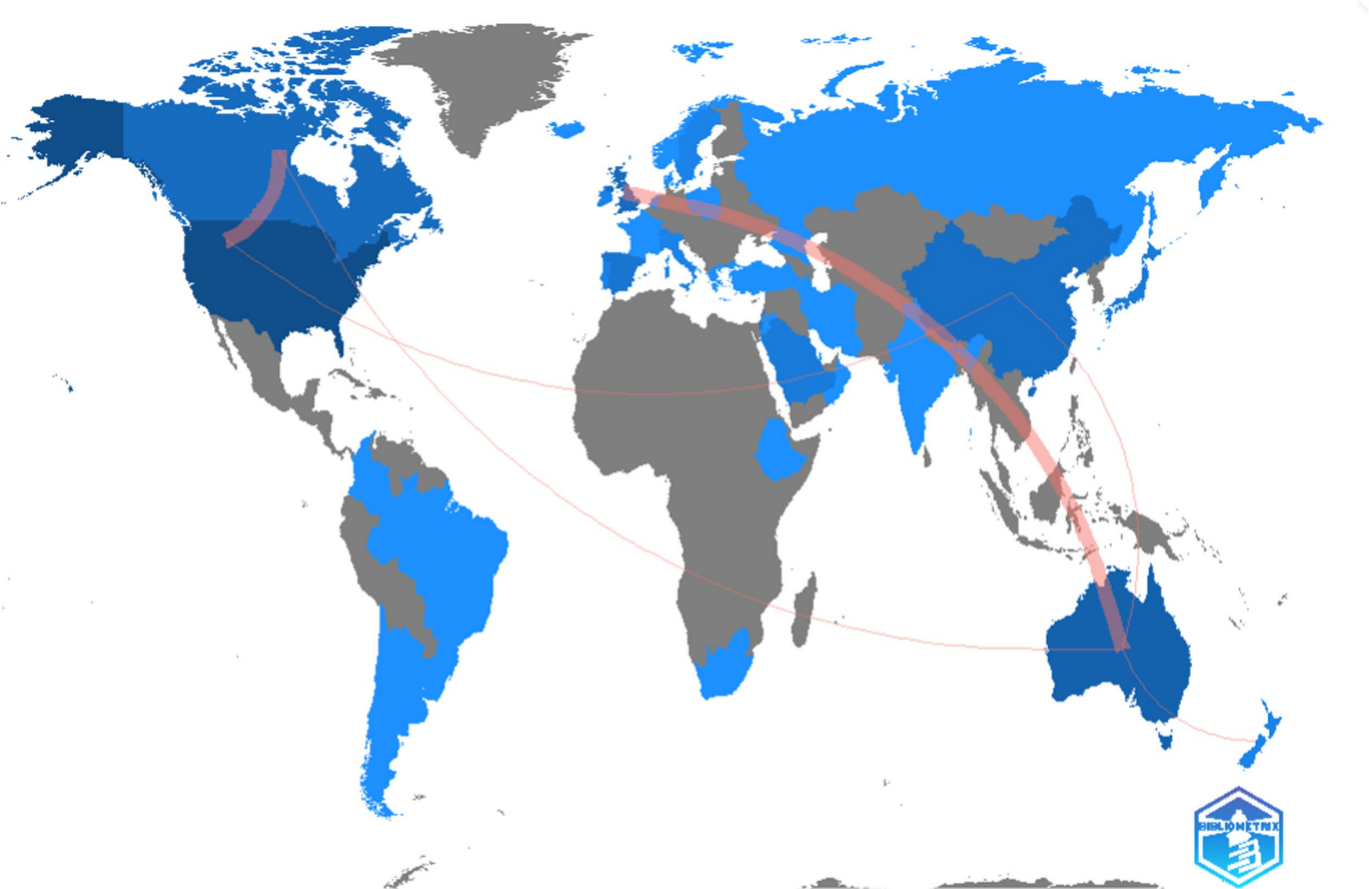
Country collaboration networks (≥1 collaboration).

The analysis of collaborations between countries shows the international relations in the research field studied. Figure 10 shows the collaborations between countries, and in particular collaborations between the United States, Canada, Great Britain and Australia.

### 3.3 Thematic analysis

The analysis of bibliographic coupling by documents involves examining shared bibliographic references among the analyzed articles. This technique helps to identify articles that have references in common, suggesting a thematic or conceptual relationship between them.

**Figure 11:**
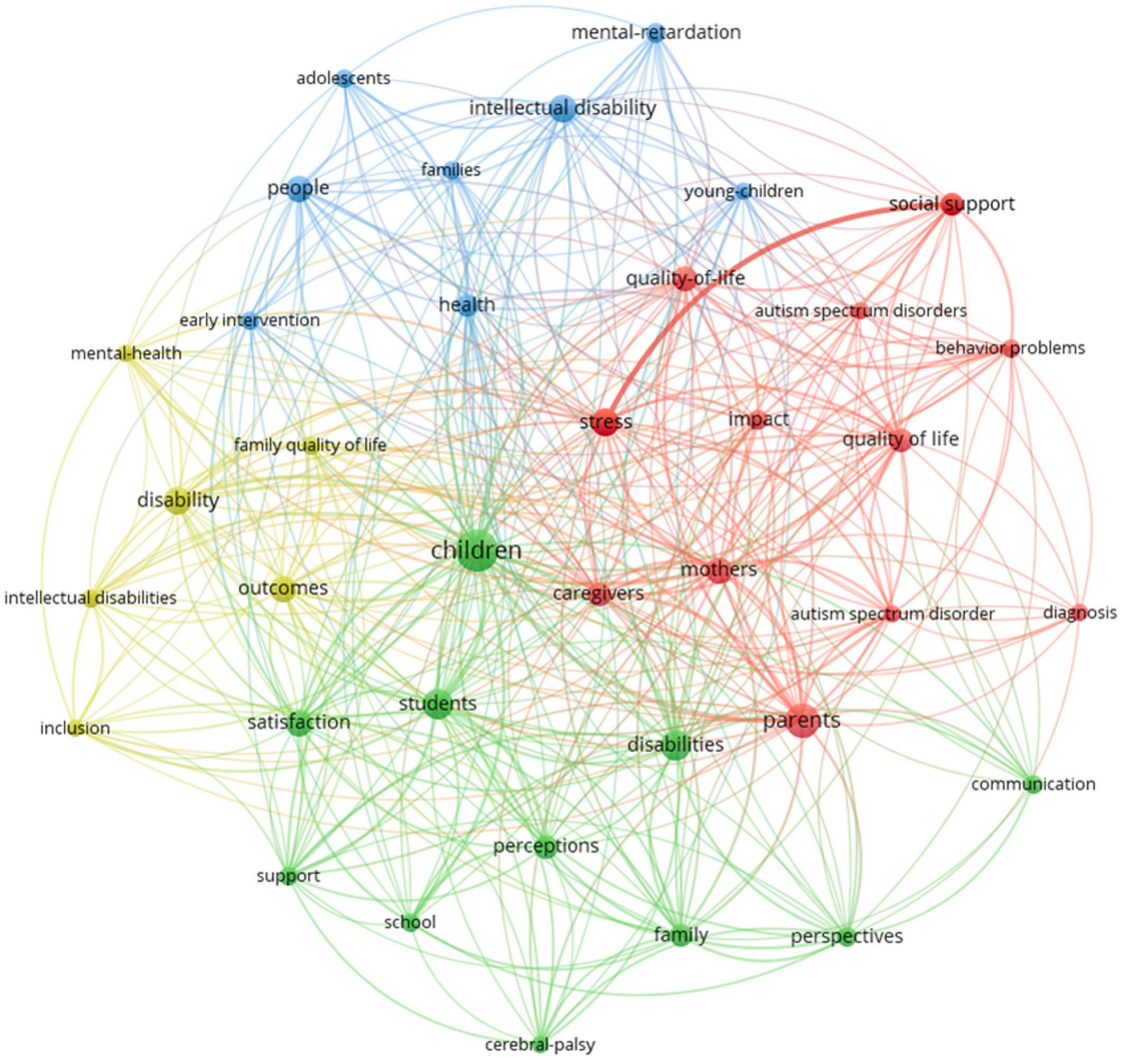
Bibliographic coupling analysis for co-word networks (≥5 co-word networks).

In this analysis, a criterion of at least 10 citations per document was established to consider the connection. Subsequently, the connected documents were selected, resulting in a total of 30 documents grouped into seven different clusters, each represented by a color in Figure 11. The size of the text in the visualization is proportional to the number of citations received and the frequency of connections between them. This graphical representation enables you to identify thematic groups and their level of interconnection within the field of study being analyzed.

In summary, this approach assists in understanding the structure and relationships within the document set, providing a clearer view of thematic areas and their degree of interrelation in your research field.

#### 3.3.1 Bibliographic coupling by document and keyword

The coexistence of bibliographic references in the articles analyzed is examined in the analysis of bibliographic coupling by documents. This helps to identify articles that share common references, indicating a thematic or conceptual relationship between them.

**Figure 12:**
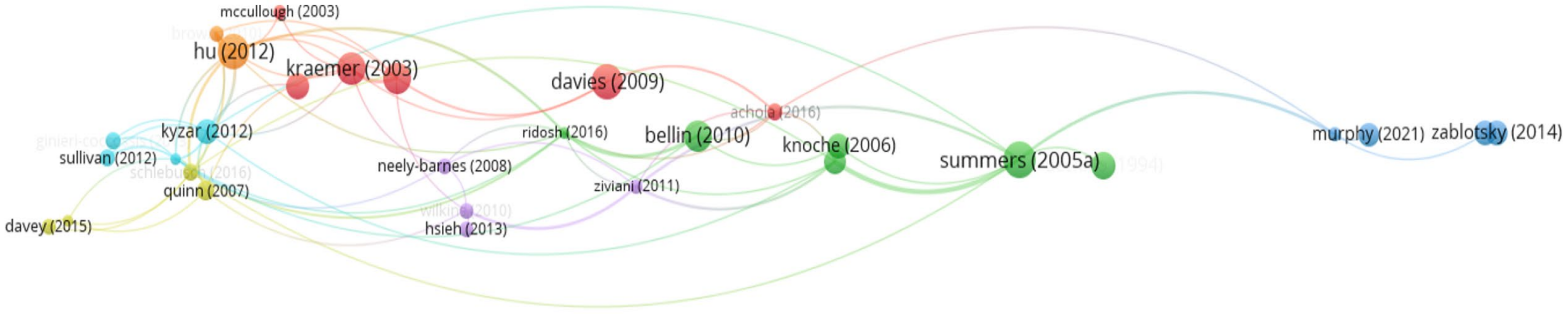
Bibliographic coupling analysis for documents (≥10 citations of publications).

The analysis was performed using a cut-off point of at least 10 citations per document. The documents that were connected were then selected, resulting in a total of 30 documents distributed in seven different clusters, which are each represented by a color in Figure 12. The size of the text displayed is proportional to the number of citations received and the frequency of connections between them. This visual representation shows the thematic groups and their level of interconnection in the field of study analyzed.

We present below a thematic review of each of the clusters, together with the number of papers, citations and the most important authors.

*Cluster 1* – *Red (293 citations, 6 papers)*: Importance of family participation during the schooling of people with intellectual disabilities and its impact on the quality of life of all family members.

This group of papers, consisting of 6 studies, received a total of 293 citations. Its subject matter focuses primarily on the importance of families’ participation in the schooling of their children with disabilities in order to design and establish educational pathways.

One of the most extensively cited articles, written by Davies and Beamish (2009), received 79 citations. In their study, the authors highlight the parents’ perspective on the planning of educational pathways for their children with intellectual disabilities, emphasizing the importance of the family’s participation in establishing educational goals during schooling. Likewise, the results of the study show the significant impact of the transition to post-compulsory education on the life of the entire family, and they express concern about the challenges that arise at the end of schooling.

The second most frequently cited articles were those by Kraemer et al. (2003) and the article by Neece et al. (2009), both of which had 63 citations.

The former examines the quality of life and satisfaction of young people with moderate or severe intellectual disabilities by means of Schalock and Keith’s Quality of Life Questionnaire (QOL-Q) (1993). The study relates the variables that make up family satisfaction with the indicators of quality of life, and finds a significant relationship between the satisfaction shown by young people and the indicators related to their family and environment. Meanwhile, the paper by Neece et al. describes the transitional period for young people with intellectual disabilities as a stressful time for their families, and suggests the need to consider the broader family when planning for the transition to adulthood during schooling.

Another important article within this cluster is the study by Al-Krenawi et al. (2011) with 46 citations. The authors discuss the strong impact of caring for a child with intellectual disabilities, and show how families report poorer family functioning, lower satisfaction, poorer quality of partnership and a more limited sense of family coherence. They therefore conclude that it is important to take these factors into account in educational services and public education when dealing with a student with an intellectual disability, and they emphasize the family-centered approach to educational planning.

*Cluster 2* – *Green (297 citations, 6 papers)*: Support for young people with special educational needs. The importance of mental health and the family relationship.

The green cluster contains 6 articles that received 297 citations, and which focus on measuring mental health and associated variables among primary caregivers caring for children with special educational needs. These articles highlight the importance of the family’s mental health as a protective factor for the well-being of children, and its positive influence on the satisfaction of the family as a whole.

The most extensively cited article in this group, with 89 citations, is by Summers et al. (2005a,b). In this study, the authors consider the importance of psychometrically measuring the quality of the relationship that is established between families and education professionals. They discuss the development of the Family-Professional Partnership Scale, which evaluates families’ perception and satisfaction with their relationships of partnership with the professionals who deal with their children with intellectual disabilities. They also state that the scale is a reliable instrument that provides an acceptable assessment of the quality of those relationships.

The second most cited paper in the green cluster, with 64 citations, is by Bellin et al. (2010). This article addresses the relationship between satisfaction with family functioning and psychological and emotional well-being, and highlights the importance of psychosocial support and the family system as a protective factor for young people with disabilities. The protective influence of family cohesion and adolescent satisfaction with their functioning is discussed.

Finally, there is the article by DeChillo et al. (1994), which is the third most cited with 50 citations. This study focuses particularly on the relationship between professionals and families when the children also suffer from emotional disorders. In their study, they identify four significant dimensions to be taken into account, which are the supportive relationships that are established, the agreements reached, a forthright exchange of information, and a flexible and shared approach in the cooperation between professionals and families.

*Cluster 3* – *Blue (139 citations, 4 papers):* Satisfaction and access of families to educational support services.

The blue cluster is in third place. It is composed of four articles, and has received 139 citations, and its main theme is access to support services for families with children with special educational needs, as well as their satisfaction with those services.

The most cited article is the paper by Murphy et al. (2021) with 46 citations. The authors investigate the impact of the pandemic on the provision of services to people with special educational needs and their families’ satisfaction with those services. The results reveal a low level of satisfaction of the families with the services received during the pandemic, but a positive attitude toward online care. For this reason, the article advocates telecare as a promising alternative for providing high-quality services in situations that may develop in a manner similar to the COVID-19 pandemic, and for situations in which families may face other barriers to accessing educational support services.

The article by Alfuraydan et al. (2020), with 45 citations, is the second most cited in this cluster. Based on the previous line of study, the authors conclude that the use of telecare and videoconferencing can improve care for families who have children with special educational needs. The results show that the use of these technologies has acceptable results for providing training and support for families, and these results are consistent with the families’ description of them as acceptable, effective and usable systems.

The third most cited article in this cluster is by Zablotsky et al. (2014), with a total of 44 citations. The authors highlight the need for accessible services for young people with disabilities and their families, and emphasize the impact this can have on the family’s socio-economic situation.

*Cluster 4* – *Yellow (81 citations, 4 papers)*: Family perception of therapies and intervention and educational support programmes.

The yellow cluster is composed of 4 articles that received a total of 81 citations. The articles focus primarily on an assessment of the support services and programmes available to families of children with disabilities.

The most cited article is by Quinn et al. (2007) with 28 citations. The authors evaluate the effectiveness of a training programme for families with school-age children with intellectual disabilities. The programme includes various types of training, which combine watching videos with modeling measures to be followed. Based on the evaluation, in the results the authors present the positive assessment of this type of training by the parents, who consider this intervention to be effective and valuable, especially for families with children with intellectual disabilities. They also saw improvements in aspects of their family life after the training.

The second most cited paper in the yellow cluster is by Schlebusch et al. (2016), which received 24 citations. The study focuses on South Africa, and evaluates the services received by families with children with disabilities. It considers the family to be a complex and interactive social system, in which all the members are connected to each other and to their environment. The scarcity of resources and support programmes is evident in the results, and is a challenge that is met by the families, which are mainly composed by a single parent, most of whom are women. The importance of understanding the families’ circumstances and daily activities if professionals are to plan and implement supportive interventions is emphasized. Any support services offered to families who have children with intellectual disabilities should foster a sense of competence and confidence in managing their daily lives.

Finally, there is the third most cited article by Davey et al. (2015) which has 20 citations. In their study, the authors highlight the importance of providing considerable resources and supports for the family targeted at financial resources to increase satisfaction and facilitate social participation. They describe the need for flexible support adapted to the families’ needs and preferences, while also highlighting their ability to choose from the resources available to them.

*Cluster 5* – *purple (75 citations, 4 articles)*: Importance of quality of life and family satisfaction with participation and access to educational and support services.

The purple cluster, in fifth place, is composed of four articles that received a total of 75 citations. These articles focus on the significant relationship between family satisfaction and perceived quality of life, with the greater involvement and access to support services that families have.

The most cited article is by Hsieh et al. (2013) with 21 citations. The authors investigate the quality of life of children with disabilities and their families, and the impact this can have.

Their results show how the quality of life and satisfaction reported by families of children with disabilities influence the children’s cognitive development and overall functioning, which has practical implications for educational services and programmes.

The studies by Neely-Barnes et al. (2008) and Wilkins et al. (2010) received 20 citations. Neely-Barnes et al. highlight the involvement of families with children with disabilities with the services as a key factor in intervention, and show how greater involvement by the family leads their children receiving more services, and higher levels of satisfaction as a result. Wilkins et al. (2010) establish a relationship between the satisfaction of families with children with disabilities and more respectful and supportive care from services, and greater family involvement and participation.

*Cluster 6* – *sky blue (98 citations, 4 articles)*: Functioning and adaptation of families with children with disabilities. Support networks.

The sixth cluster is composed of four articles that received a total of 98 citations. These articles focus on the functioning and adaptation of families with children with disabilities.

The most frequently cited article in this cluster, with 45 citations, is by Kyzar et al. (2012). The authors conduct a study that addresses the gradual shift in the understanding of disability from a deficit-centered model to a support-based model. They emphasize the fact that although more attention is being paid to supporting children with disabilities, the support needs of their families are increasing. This research underscores the importance of taking these needs into account in policy and in all areas of management, including education, as families with children with severe and multiple disabilities may also be at greater risk of having unmet needs.

Sullivan et al. (2012) conducted a study that received 23 citations. The authors investigate the relationship between the presence of a child with a disability in the family and how the family functions in broader terms, while also considering the important role of other figures such as grandparents as a natural resource providing support. They conclude with the importance of including grandparents in meetings to facilitate support for families who have children with disabilities.

In the study by Ginieri-Coccossis et al. (2013), which received 21 citations, the parents report lower levels of satisfaction in the areas of social relationships and environment. This was also shown to be a predictor of the emotional well-being and school functioning of their children with intellectual disabilities.

*Cluster 7* – *orange (97 citations, 2 articles)*: Quality of life of families with children with disabilities.

Finally, there is the orange cluster, which has a total of 97 citations and is composed of two articles. Both papers address the quality of life of families with children with disabilities and its importance in the organization of support and intervention structures.

First, there is the study by Brown et al. (2010), which received 81 citations. In this book chapter, the authors examine the satisfaction with their quality of life of families who have children with disabilities. In their research, they describe different variables and factors that contribute to a better understanding of the family’s quality of life, and the importance of taking them into account. The variables described include the family’s age, place of origin and the support they need. They emphasize that due to the variety in types of families, in order to determine their quality of life and to be able to propose flexible support strategies, it is necessary to ascertain both the internal and the external aspects of the family’s life, in order to foster an optimal quality of family life and therefore help the child with disabilities to adapt.

The second article in the orange cluster is by Hu et al. (2012). This study highlights the growing importance of family quality of life in the field of intellectual disability as an important framework for assessing the support needs and services families should receive. The authors identify variables such as living conditions (e.g., housing and transportation), family income, and the severity of the disability as significant predictors of how families perceive their quality of life.

A bibliographic coupling for co-word networks was then performed, and a group of four clusters of different colors is shown in Figure 11.

Four main groups of keywords were identified, with the size of the text displayed being proportional to the frequency of its occurrence and the number of connections between them. The cut-off point was set at 5 or more occurrences of these keywords, and there were 37 of them.

The first group is composed of 12 keywords, focusing on families with core terms such as “parents,” “mothers” and “caregivers.” It also includes related and connected concepts such as “quality of life,” “stress,” “impact” and “social support.”

The second group consists of a network composed of 11 words with “children” as the central point and “students,” “satisfaction,” “disabilities,” “perceptions,” “school” and “family” as the most important connected concepts.

The third network is composed of 8 keywords, with “intellectual disability” and “health” as the central terms, which are related to others including “early intervention,” “mental-retardation and “young-children.”

In the last group, with 6 keywords, “disability,” “outcomes” and “family quality of life” are the central terms. It refers to concepts such as “mental health” and “inclusion.”

#### 3.3.2 Strategic thematic analysis

The study was based on bibliometric analysis conducted using the R bibliometric software package, renowned for its effectiveness in evaluating and visualizing bibliometric data. This software offers statistical tools and algorithms to identify relevant patterns and trends in academic literature. Among its features, it highlights the generation of strategic diagrams, which provide a clear visual representation of the thematic structure of the research field. These diagrams, divided into quadrants, allow the classification of topics according to their relevance and degree of development. The interpretation of these diagrams is based on the position of key terms, facilitating the identification of main, specialized, emerging, or disappearing topics.

**Figure 13:**
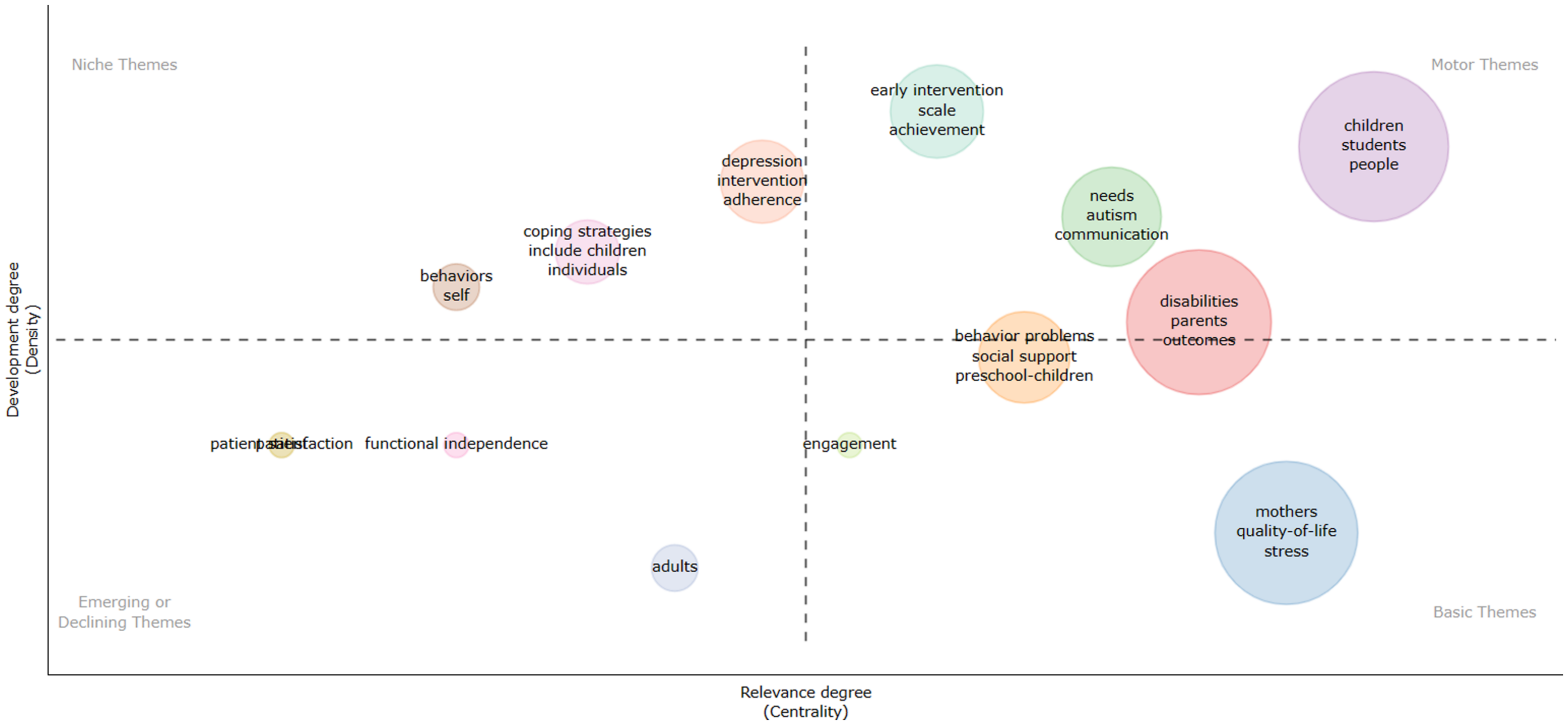
Strategic diagram of family satisfaction with educational inclusion.

Figure 13 summarizes the themes addressed in this study. In this diagram, the size of the spheres is directly related to the frequency of occurrence of the keywords. The areas of the diagram are divided into quadrants for easier understanding: the upper right quadrant represents the main or driving topics; the upper left quadrant, highly specialized or niche topics; the lower right quadrant, fundamental or basic topics; and the lower left quadrant, emerging or disappearing topics. This visual representation allows for a clear understanding of the distribution and relative importance of the different topics identified in the bibliometric analysis.

In the upper right quadrant are topics such as “children,” “needs,” “early intervention,” and “disabilities,” which are highly relevant and have a solid development in the research field. On the other hand, the upper left quadrant harbors highly specialized but marginally important topics for the present study, such as “depression,” “coping strategies,” and other related topics. These topics show solid internal connections but low external relevance.

Topics in the lower left quadrant, such as “functional independence” and “patient satisfaction,” seem to be situated at an intermediate point, suggesting they could become emerging topics due to their centrality in the study. Meanwhile, the lower right quadrant contains essential but still developing topics, such as “behavior problems,” “social support,” and others related. These topics are crucial for the research field, though they require more attention and development.

In summary, the thematic analysis indicates that a research focus on topics such as “behavior problems,” “social support,” and “preschool-children” could yield significant results, as these topics are fundamental but still in development and show good centrality in the study.

## 4 Discussion

This article presents a comprehensive bibliometric study which examines the scientific production on the satisfaction that families with children with intellectual disabilities have as regards educational inclusion. The analysis identifies emerging trends and distinctive characteristics of research related to the topic studied, and the results extracted provide a solid foundation for implementing strategies for improvement in educational institutions, with the ultimate aim of optimizing the quality of education.

The main findings obtained therefore provide relevant data. The analysis reveals a steady increase in the number of articles published during the period studied, from 1993 to 2022. Initially, a single publication was recorded in 1993, marking the beginning of research in this field. However, there has been a significant albeit fluctuating increase in the number of articles published since 2003, when the number of annual publications was surpassed, with a total of four publications. Despite the annual variations in the number of articles, the general trend is clearly upward, indicating a growing interest in and relevance of the subject of study, mainly from 2015 onwards. It is also interesting to note that the World Education Forum took place in the Republic of Korea in that year, and its conclusions were included in the Incheon Declaration, in which inclusive education is one of the objectives of the framework for action established by all UNESCO’s (2015) States Parties.

Focusing on the results after the authors’ analysis of the satisfaction of families of students with disabilities in relation to educational inclusion, the study shows that a total of 349 researchers participated in the scientific production on this topic. Among the most prominent are Summers JA and Blacher J, who made significant contributions to the scientific literature with 3 papers each, and received 172 and 145 citations, respectively.

In terms of impact and recognition, the leading authors are responsible for many global citations, and the TGCS cut-off point is established at 129.

As for the results for the most important institutions, although several universities are significant in terms of their research production in the area studied, the University of Kansas is the leader both in the number of documents published, with a total of 4, and in global citations received, with a total of 250. In addition, universities in California, and more specifically, the University of California at Los Angeles and Riverside, have also contributed significantly to the scientific literature in this field, with a considerable number of global citations, at 163 and 145, respectively.

Likewise, the results obtained reveal interesting patterns of scientific production and the influence of countries. In terms of the number of publications, the United States stands out with 36 articles, followed by Australia with 12 publications. Other countries identified were Canada and the United Kingdom, with 7 articles each. However, if the number of global citations received is considered, although the United States and Australia continue to lead with 964 and 184 respectively, China is in third place, as despite having three publications identified, it has many citations, with 97 TGCS, placing it ahead of Canada and the United Kingdom.

An analysis of the journals that have made the greatest contribution to the dissemination of scientific literature in the field of research provides valuable information, as the most prominent in terms of the number of articles published is the “Journal of Intellectual Disability Research,” which heads the list with four articles. It is followed by “Child Care Health and Development,” “Exceptional Children” and “Frontiers in Education,” with two articles. As for the impact factor, “Exceptional Children” stands out among these three journals with an impact factor of 4.09, placing it in the first quartile.

The collaboration networks between authors are also interesting, as the main network composed of five authors involves close collaboration and ongoing work. The authors in the network include Summer JA, one of the most important authors in the area of scientific production in our study, as mentioned above, as well as Poston D., Hoffman I., Marquis J and Turnbull A.

The thematic analysis carried out shows that actions aimed at students with special educational needs and their families are also relevant issues to be taken into account in the design of educational support. According to the literature, the studies reviewed emphasize the importance of the family’s participation in the life of the school and their cooperation with the educational team as key factors in improving family satisfaction and therefore contributing to educational inclusion (Kraemer et al., 2003; McCullough and Huebner, 2003; Davies and Beamish, 2009; Neece et al., 2009; Al-Krenawi et al., 2011; Achola and Greene, 2016). The authors stress the importance of establishing effective channels for communication between families and schools, as well as involving families in decision-making about their children’s education.

In line with the literature review, other authors mention the importance of offering accessible educational support strategies to families with children with disabilities, and highlight the use of telecare and new technologies as means to develop flexible support measures that adapt to the new family circumstances (Zablotsky et al., 2014; Slade et al., 2018; Alfuraydan et al., 2020; Murphy et al., 2021). These results can also contribute to the issues mentioned above, facilitating the creation of effective communication channels.

Another issue to highlight, which is consistent with the results of the thematic analysis and the literature reviewed, is the significant relationship observed between the families’ satisfaction with the educational support services they receive and the quality of life they claim to have. The authors also mention the influence this factor has on the cognitive and emotional development of their children with disabilities (Neely-Barnes et al., 2008; Wilkins et al., 2010; Ziviani et al., 2011; Hsieh et al., 2013).

It is essential to consider all of these findings in order to contribute to the fulfillment of current international education guidelines focused on ensuring inclusion and quality education for all students. In addition to the natural support network that families with children with disabilities need, the educational institution is a crucial actor in enhancing the quality of life of these families, and is a fundamental means of support (Kyzar et al., 2012).

## 5 Conclusion

The main objective of this comprehensive bibliometric study was to investigate the satisfaction of families with children with intellectual disabilities in the context of inclusive education. Based on this objective, detailed analyses were conducted on the scientific production related to this topic, aiming to provide a deep and updated understanding of the interactions between family satisfaction and inclusive educational practices. The results obtained not only inform about the current state of the field but also offer valuable insights for future research and the development of more effective educational policies.

This exhaustive bibliometric study on family satisfaction with children with intellectual disabilities in the context of inclusive education has yielded significant results with direct implications for the educational and academic sphere. The increasing trend in the number of publications from 1993 to 2022, with a marked rise from 2003 onwards and a critical point in 2015 coinciding with the World Education Forum, underscores the global interest in this topic. The United States leads in terms of academic production, followed by Australia, although China shows emerging influence in this field.

The study identifies the fundamental role of key researchers and prominent journals such as the “Journal of Intellectual Disability Research” and “Exceptional Children” in advancing knowledge in inclusive education and family satisfaction. It emphasizes the need to actively involve families in the education of their children with disabilities, promote effective communication between families and educational institutions, and offer accessible support strategies, including the use of technologies.

It is concluded that educational institutions play a crucial role as support networks for families with children with disabilities, emphasizing the importance of family collaboration, flexible support measures, and joint decision-making. An inclusive educational response not only enhances family quality of life but also benefits the cognitive and emotional development of children with disabilities.

This study identifies important research areas and provides a comprehensive view of the field of inclusive education and family satisfaction. It highlights the need to consider family perspectives, address the support needs of children with disabilities, promote inclusion in educational environments, and ensure the overall well-being of students and their families.

The significance of this study lies in its ability to provide a deep and updated understanding of the interaction between family satisfaction and inclusive education for children with intellectual disabilities. By identifying trends, patterns, and key research areas, this analysis not only informs about the current state of the field but also points toward future directions for research and educational practice. It underscores the influence of key researchers, prominent journals, and leading countries, providing a solid foundation for international collaboration and progress toward high-quality inclusive education for all children. In summary, this study not only expands existing knowledge but also serves as a guide for the development of policies and practices that promote more effective and needs centered inclusive education for families and students with intellectual disabilities.

For future lines of research, this study opens up several interesting opportunities. One possible research area could focus on further exploring the specific impact of different family support strategies on the satisfaction and well-being of children with intellectual disabilities in inclusive educational settings. Additionally, it would be beneficial to investigate how national and international educational policies can be adapted to enhance collaboration between families and educational institutions, as well as to provide better access to resources and support services.

On the other hand, it is important to acknowledge the limitations of this study. Despite its comprehensiveness, this bibliometric analysis may be biased toward certain geographical regions or publication languages, which could influence the representativeness of the results. Furthermore, since it is based on bibliometric data, some dimensions of family satisfaction and inclusive educational practices may not be fully captured. Therefore, future research could benefit from mixed methods approaches that combine quantitative and qualitative methods to obtain a more comprehensive and contextualized understanding of these phenomena.

In terms of benefits to the reader, this article offers a comprehensive and up-to-date synthesis of the current state of the field, as well as valuable insights for future research and the development of more effective educational policies. By highlighting trends, patterns, and key areas of research, this study provides clear guidance for academics, professionals, and policymakers interested in promoting inclusive education and improving the satisfaction of families with children with intellectual disabilities.

## Data availability statement

The raw data supporting the conclusions of this article will be made available by the authors, without undue reservation.

## Author contributions

ST-Y: Investigation, Resources, Conceptualization, Data curation, Visualization, Writing – original draft. DN-M: Investigation, Formal analysis, Funding acquisition, Supervision, Writing – review & editing. MTG-D: Funding acquisition, Investigation, Supervision, Writing – review & editing, Methodology, Validation, Resources. VG-D: Conceptualization, Investigation, Methodology, Software, Validation, Visualization, Writing – original draft.
